# Insight the Biological Activities of Selected Abietane Diterpenes Isolated from *Plectranthus* spp.

**DOI:** 10.3390/biom10020194

**Published:** 2020-01-28

**Authors:** Przemysław Sitarek, Monika Toma, Epole Ntungwe, Tomasz Kowalczyk, Ewa Skała, Joanna Wieczfinska, Tomasz Śliwiński, Patricia Rijo

**Affiliations:** 1 Department of Biology and Pharmaceutical Botany, Medical University of Lodz, Muszyńskiego 1, 90-151 Łódź, Poland; przemyslaw.sitarek@umed.lodz.pl (P.S.); ewa.skala@umed.lodz.pl (E.S.); 2 Laboratory of Medical Genetics, Faculty of Biology and Environmental Protection, University of Lodz Pomorska 141/143, 90-236 Lodz, Poland; monikatoma3@gmail.com (M.T.); tomasz.sliwinski@biol.uni.lodz.pl (T.Ś.); 3 Research Center for Biosciences and Health Technologies (CBIOS), Universidade Lusófona de Humanidades e Tecnologias, 1749-024 Lisboa, Portugal; epole.ntungwe@ulusofona.pt; 4 Department of Molecular Biotechnology and Genetics, University of Lodz, S. Banacha 12/16, 90-237 Lodz, Poland; tomasz.kowalczyk@biol.uni.lodz.pl; 5 Department of Immunopathology, Medical University of Lodz, Żeligowskiego 7/9, 90-752 Lodz, Poland; joanna.wieczfinska@umed.lodz.pl; 6 Instituto de Investigação do Medicamento (iMed.ULisboa), Faculdade de Farmácia, Universidade de Lisboa, 1649-003 Lisboa, Portugal

**Keywords:** natural abietane diterpenes, cytotoxic activity, apoptosis, cell cycle, gene expression, H2A.X, mitochondrial membrane potential

## Abstract

Natural compounds isolated from plants are excellent starting points in drug design and have been widely studied as anticancer agents; they hence find use in a considerable proportion of anticancer drugs. The genus *Plectranthus* (Lamiaceae) comprises a large and widespread group of species with various applications in traditional medicine. Therefore, the aim of the present study was to determine the effectiveness of treatment with four abietane diterpenoids isolated from *P. madagascariensis* and *P. ecklonii*, 6,7-dehydroroyleanone, 7β,6β-dihydroxyroyleanone, 7α-acetoxy-6β-hydroxyroyleanone and parvifloron D, in initiating apoptosis in a glioma cell line. The pure compounds were found to exhibit anticancer effects in primary H7PX glioma cells line by inducing apoptosis G2/M cell cycle arrest and double-strand breaks, indicated by increased levels of phosphorylated H2A.X and decreasing mitochondrial membrane potential; they also influenced the expression of pro- and anti-apoptotic genes (*Bax*, *Bcl-2*, *TP53*, or *Cas-3*). Our findings indicate that these compounds may offer potential as beneficial antitumor drugs but further in vivo studies are needed.

## 1. Introduction

Glioblastoma multiforme (GBM) is the most malignant and most common form of primary astrocytoma, accounting for more than 60% of all brain tumors in adults [1]. Despite the development of new therapies, it demonstrates high mortality, with patients expecting a median survival period of approximately 14 to 15 months from diagnosis [2,3]. There is hence a pressing need to identify new compounds for use in therapy. The plant kingdom is a valuable source of natural compounds with potential anti-cancer activity: many metabolites of plant origin have a broad spectrum of activity and are less toxic to patients than synthetic drugs [4]. One potential source is represented by the plants of the genus *Plectranthus* [5].

**Figure 1:**
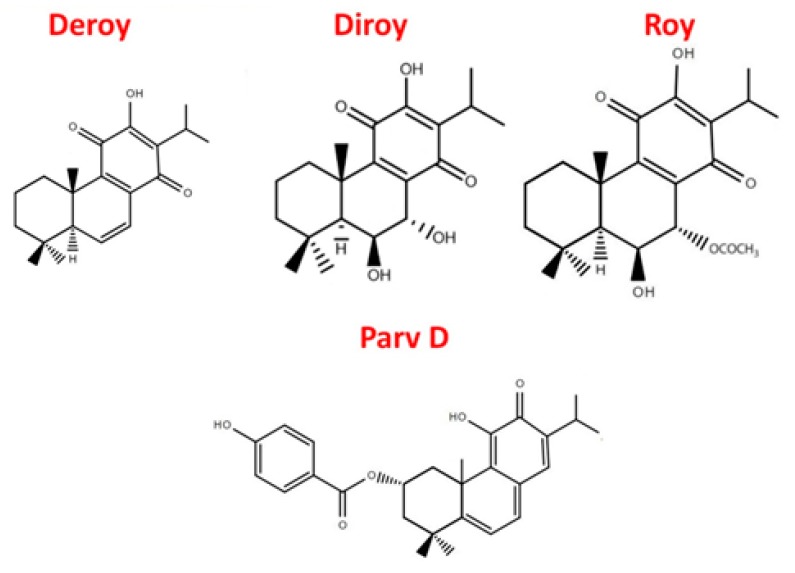
Abietane diterpenes isolated from *P. madagacariensis* and *P. ecklonii*.

The genus *Plectranthus* (Lamiaceae) is located in the tropical and warm regions of the Old World, including Africa, Australia and India [6]. Of 300 species of *Plectranthus*, 62 are reported to be used as traditional medicines, foods, ornamentals, flavorings, animal fodder and materials; however, over 85% of these species are used in medicine. *Plectranthus* is a valuable source of secondary metabolites with proven cytotoxic, antiproliferative, anti-bacterial, anti-fungal, anti-plasmatic, or anti-cancer effects [6,7]. Numerous studies attribute these biological properties to the diterpenes expressed by this genus [8,9,10]. Our previous studies have demonstrated that the diterpenes isolated from *Plectranthus* genus induce apoptosis in various cancer cell lines, including human myeloid leukaemia, and pancreatic, lung, melanoma, prostate, and breast cancer [11,12,13,14,15,16,17]. The present study focuses on the properties of abietane diterpenes (Roy, Deroy, Diroy, and Parv D) isolated from *P. madagascariensis* and *P. ecklonii.* (Figure 1).

*P. madagascariensis* can be found in regions of South Africa [18] and has been traditionally used for skin ailments, such as treatment of scabies and small wounds. It is also used for colds and asthma [6]. Similarly to other members of this genus, *P. madagascariensis* possesses a number of secondary metabolites with antimicrobial, antioxidant or anticancer properties [15,16]. The essential oil is a rich source of 6,7-dehydroroyleanone (Deroy), i.e., an abietane diterpene with a quinone moiety [15]. However, the acetone extracts are rich in various royleanones, including 7β,6β-dihydroxyroyleanone (Diroy) and 7α-acetoxy-6β-hydroxyroyleanone (Roy) [18]. Another *Plectranthus* member, *P. ecklonii*, is highly rich in another abietane diterpene, parvifloron D (Parv D), which has long been known to display potent anticancer activities [19,20].

To gain a greater insight into their potential use in cancer treatment, the present study examines the apoptotic character of these *Plectranthus*-derived abietanes and their activities against a human glioma cell line.

## 2. Materials and Methods

### 2.1. Plant Material

*Plectranthus ecklonii* and *Plectranthus madagascariensis* (Lamiaceae family) were cultivated in Instituto Superior de Agronomia Campus (Lisbon University), from seeds provided by the Herbarium of the Botanical Garden of Lisbon, Portugal. Their growth was monitored at Parque Botânico da Tapada da Ajuda from cuttings collected in June and September in the years 2007 and 2008 (provided by the Kirstenbosch National Botanical Gardens, South Africa). Voucher specimens were deposited at Herbarium “João de Carvalho e Vasconcellos” of the “Instituto Superior de Agronomia”, Lisboa (LISI), Portugal.

### 2.2. Isolation and Identification of Abietane Diterpenes

The abietane diterpene 6,7-dehydroroyleanone (Deroy) was extracted from the essential oil obtained through the hydrodistillation of *P. madagascariensis* leaves and stems in a Clevenger-apparatus, while 7β,6β-dihydroxyroyleanone (Diroy) and 7α-acetoxy-6β-hydroxyroyleanone (Roy), were isolated from acetonic extracts of the same plant. Parviforon D (Parv D) was isolated from an acetonic extract of the whole plant of *P. ecklonii*. The isolation and identification of all compounds were described earlier [15,19,21].

### 2.3. Cell Cultures

Our studies were performed on a human primary H7PX glioma cell line obtained from a cancer patient diagnosed with grade 4 glioblastoma multiforme. The study was approved by the Ethical Commission of the Medical University of Lodz, and informed consent was obtained from the patients (Nr. RNN/194/12/KE). Cell lines were maintained in an incubator with 5% CO_2_ atmosphere at 100% humidity and 37 °C in DMEM medium supplemented with 10% (*v*/*v*) heat-inactivated Fetal Bovine Serum (FBS) and 100 U/mL penicillin, 100 μg/mL streptomycin. All cell culture media and components were purchased in Lonza (Basel, Switzerland).

### 2.4. Cell Viability

MTT assay was employed to measure the viability of primary H7PX glioma cells treated with different concentrations of abietane diterpenes, Deroy, Diroy, Roy, and Parv D. Briefly, cells were seeded at 1 × 10^4^ cells per well in 96-well culture plates and left overnight. In the next step, the cells were incubated for 24 h with the test compounds over a range of concentrations: 0 (control), 0.39, 0.78, 1.56, 3.13, 6.25, 12.5, 25, 50, and 100 µg/mL. Following this, the cells were incubated with 0.5 mg/mL of 3-(4,5-dimethylthiazol-2-yl)-2,5-diphenyl tetrazolium bromide (MTT) at 37 °C for 1.5 h. After this time, MTT was carefully removed and DMSO (100 μL) was added to each well; the mixture was mixed at low speed for 5 min to fully dissolve the formazan crystals. Absorbance was measured at 570 nm with a reference at 630 nm using a Bio-Tek Synergy HT Microplate Reader (Bio-Tek Instruments, Winooski, VT, USA). All experiments were repeated in triplicate. Cell viability was expressed as a percentage relative to the untreated cells, which was defined as 100%. Cell line pictures after treatment were captured using HDCE-X5 digital microscope camera and Scopelmage9.0 software.

### 2.5. Apoptosis/Necrosis and Cell Cycle Detection by Flow Cytometry

In this experiment, a population of apoptotic and necrotic cells of the primary H7PX glioma cell line was detected using an annexin V-fluorescein isothiocyanate (FITC)/propidium iodide (PI) (BD Biosciences) detection kit according to the manufacturer’s protocol. Briefly, the cells were plated into 6-well culture dishes (2 × 10^5^ cells/well) for 24 h prior to the addition of all tested compounds used in this study. Following 24-h incubation with all compounds, the percentage of apoptotic/necrotic cells was determined by the annexin V-FITC/PI assay. The proportions of the H7PX cell line in different cell cycle phases was assessed using PI/RNase staining buffer (BD Biosciences). Cells were seeded as mentioned above and treated with IC_50_ concentrations of Deroy, Diroy, Roy and Parv D. After 24-h incubation, the medium was discarded, and the cells were collected and fixed with 70% cold ethanol for at least one hour at −20°C. In the next step, the cells were suspended in staining buffer. Cell analysis was performed using CytoFlex Beckman Coulter.

### 2.6. ELISA Measurement of γ-H2A.X

The primary H7PX glioma cell line was cultured at a density of 1 × 10^4^ cells per well with vehicle or with the following abietane diterpenes: Deroy, Diroy, Roy and Parv D. They were administered at the IC_50_ concentration on black 96-well plates with a clear bottom. The level of phosphorylated H2A.X histone was determined using an H2A.X Phosphorylation Assay Kit (Millipore, Billerica, MA, USA) according to the manufacturer’s protocol. Briefly, chemiluminescence detection was performed using a GloMax-Multi device (Promega). The mixtures were incubated for 30 min with 35 μM. Bleomycine as a positive control.

### 2.7. RNA Isolation and RT-PCR

The cells were plated into 6-well culture dishes (3 × 10^5^ cells/well) for 24 h prior to the addition of Deroy, Diroy, Roy, and Parv D. The total RNA isolation kit (A & A Biotechnology) was used to isolate total RNA from cells treated with the compounds at the IC_50_ value. The obtained RNA was transcribed into cDNA using TranScriba Kit (A&A Biotechnology). Following this, the expression of four genes (*Bax*, *Bcl-2, Cas-3, TP53*) was measured by qRT-PCR using TaqMan^®^ Real-Time PCR Master Mix (Life Technologies, Carlsbad, CA, USA) and Agilent Technologies Stratagene Mx300SP working on MxPro software Santa Clara, CA, USA). TaqMan probes (Life Technologies) were used to analyse genes and *18S rRNA* (Life Technologies) was included as a reference gene. The PCR was performed as follows: 95 °C for 10 min, 40 cycles of 95 °C for 15 s and 60 °C for 60 s.

### 2.8. Mitochondrial Membrane Potential (MMP)

Mitochondrial membrane potential was determined by the fluorescent probe JC-1 (5,6,6-tetrachloro-1,1,3,3-tetraethylbenzimidazolylcarbocyanine iodide). Briefly, cells were seeded into black 96-well tissue culture plates with transparent bottom (Greiner Bio-One, Monroe, NC, USA) at a density of 1 × 105 cells/well (primary H7PX glioma cells) in 50 µL culture medium and allowed to grow overnight. The next day, cells were treated with indicated concentration IC_50_ of Deroy, Diroy, Roy and Parv D for 24 h. Finally, the cells were preincubated with 5 µM JC-1 in the HBSS in a CO^2^ incubator at 37 °C for 30 min. Prior to measurements, the cells were centrifuged (300× *g* for 10 min at 22 °C) then washed twice with the HBSS. Further procedure has been described previously [22,23].

### 2.9. Statistical Analysis

Data are presented as the mean ± standard error (SD) of the mean of at least three independent experiments. The Shapiro–Wilk test was used to determine the normality of the distribution of results. The Kruskal–Wallis test with multiple comparisons of average ranks and the one-way analysis of variance (ANOVA) with the Tukey post hoc test were used to determine differences between samples. Statistica 13 software was used for all calculations. * *P* < 0.05 was considered to indicate a statistically significant difference.

## 3. Results

### 3.1. Cytotoxic Activity

**Figure 2:**
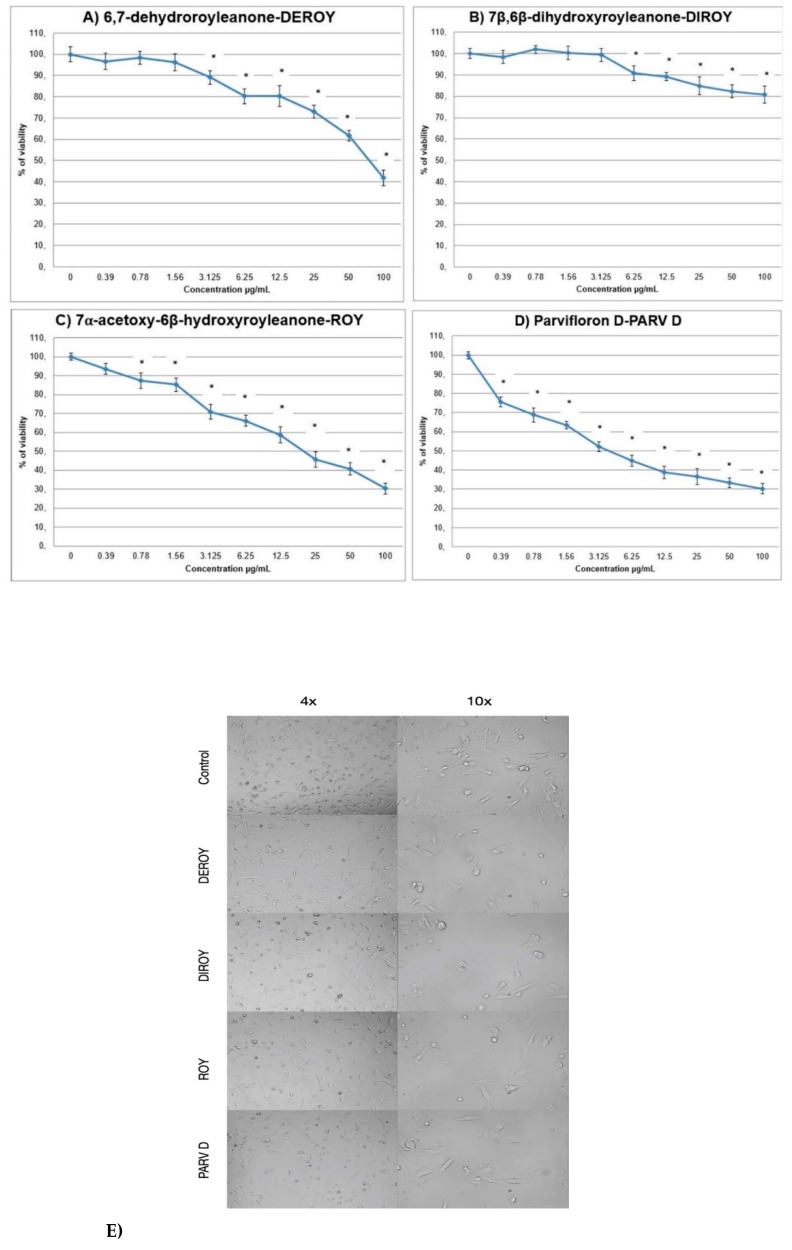
(**A**–**E**) The effect of Deroy, Diroy, Roy, and Parv D treatment on cell viability after 24-h incubation. The primary H7PX glioma cells were treated with different concentrations of these compounds. The y-axis shows the percentage of cell viability. Data are reported as means ± SD of three determinations. * *P* < 0.05 vs untreated cells E. Representative photos showing primary H7PH glioma cells after 24 h treatment with IC50 concentrations of given compounds at different magnifications 4× and 10×. Photos were captured with HDCE-X5 digital microscope camera using ScopeImage9.0 software.

The primary H7PX glioma cells were treated with the four compounds (6,7-dehydroroyleanone-Deroy, 7β,6β-dihydroxyroyleanone- Diroy, 7α-acetoxy-6β-hydroxyroyleanone-Roy and parvifloron D-Parv D) at concentrations of 0–100 µg/mL for 24 h, and the percentage of viable cells was determined by MTT assay. It was found that all tested compounds reduced the viability of human H7PX cells but to different degree: the H7PX cells were most sensitive to Parv D, Roy and Deroy compared to Diroy in the tested range of concentrations (Figure 2A–D). Figure 2E show primary H7PH glioma cells after 24 h treatment with IC50 concentrations of compounds at different magnifications.

### 3.2. Analysis of Apoptotic/Necrotic and Cell Cycle Distribution

**Figure 3:**
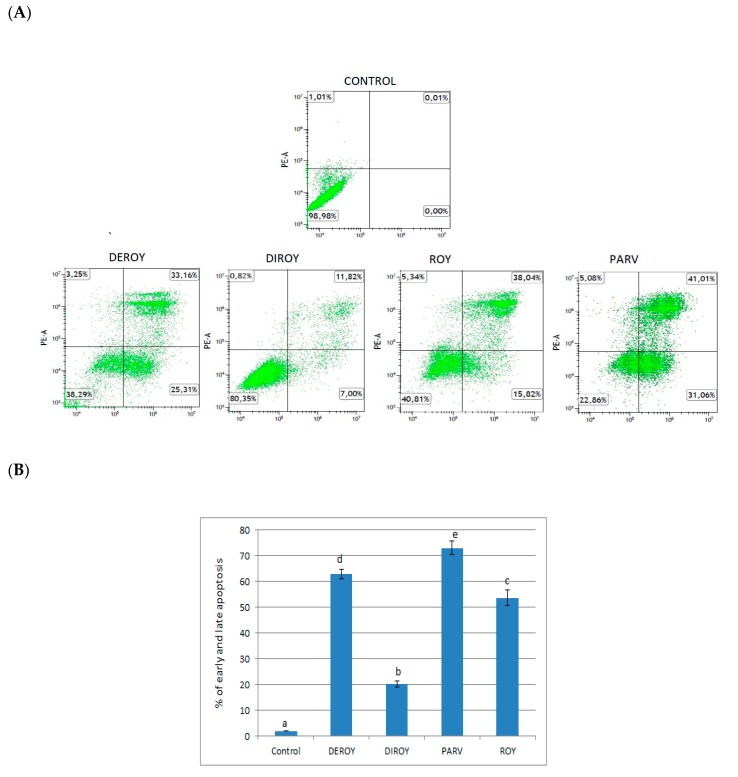

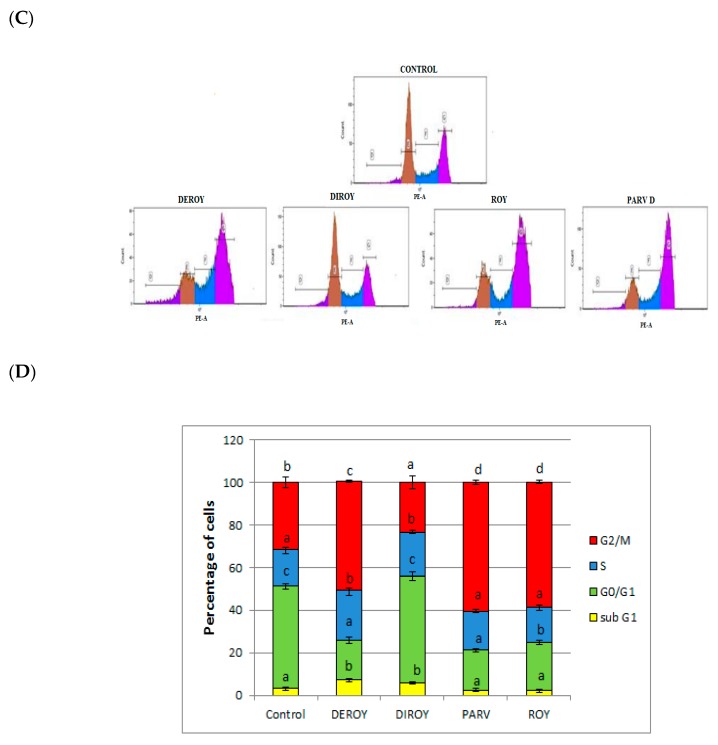
(**A**) Representative histograms of apoptosis/necrosis analysis of primary H7PX glioma cells after 24-h treatment with abietane diterpenes (**B**) The percentage of primary H7PX glioma cells in early and late apoptosis after 24-h treatment. (**C**) Representative histograms of primary H7PX glioma cell cycle distribution after 24-h treatment (**D**) The percentages of individual cell cycle phases after 24-h treatment. The values represent mean ± SD of three independent experiments. Different letter indicates differences at the P<0.05. In all figures, Deroy (6,7-dehydroroyleanone), Diroy (7β,6β-dihydroxyroyleanone), Roy (7α-acetoxy-6β-hydroxyroyleanone) and Parv D (parvifloron D).

Flow cytometry analysis was performed to verify whether the tested compounds induced apoptosis or necrosis and whether they resulted in an altered cell cycle distribution. The experiment showed that cell death after treatment occurred mainly through the apoptosis pathway. A greater percentage of early and late apoptosis was observed after treatment with Deroy, Diroy, Roy, and Parv D) (62%, 20%, 53%, and 73%, respectively) compared to untreated cells. Cell cycle distribution analysis revealed increased H7PX cell percentage in G2/M phase after treatment with Deroy, Diroy, Roy, and Parv D compared to the untreated cells, with the respective percentages being 51%, 23%, 58%, and 60% (Figure 3A–D).

### 3.3. The Level of Phosphorylated H2A.X in H7PX Cells after Treatment of Abietane Diterpenes

**Figure 4:**
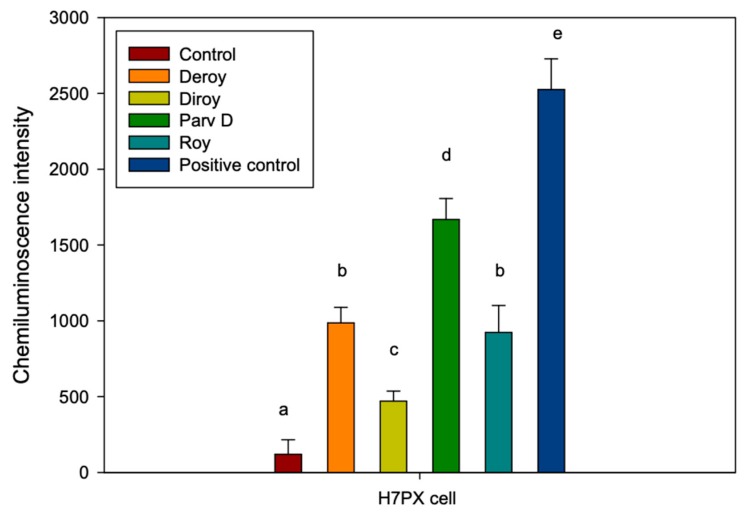
The levels of phosphorylated H2A.X identified in H7PX cell line treated with 6,7-dehydroroyleanone (Deroy), 7β,6β-dihydroxyroyleanone (Diroy), 7α-acetoxy-6β-hydroxyroyleanone (Roy) and parvifloron D (Parv D), indicating the presence of DSBs. Cells were treated with all compounds for 24 h. The mean values ± SD of γ-X2A.X were calculated from three independent ELISA experiments. Different letters indicate significant differences at *P* < 0.05. Bleomycine was used as a positive control.

Increased levels of phosphorylated H2A.X, which marks indicating the presence of DSBs (double strand breaks), were observed in cells treated with the diterpenes in comparison to untreated control cells. Significant increase in the amount of phosphorylated H2A.X histone was visible after treatment with Deroy, Roy, and Parv D. The effect after treatment with Diroy was mediate (Figure 4).

### 3.4. Determination of the mRNA Expression of TP53, Bax, Bcl-2, and Cas-3 using RT-PCR

**Figure 5:**
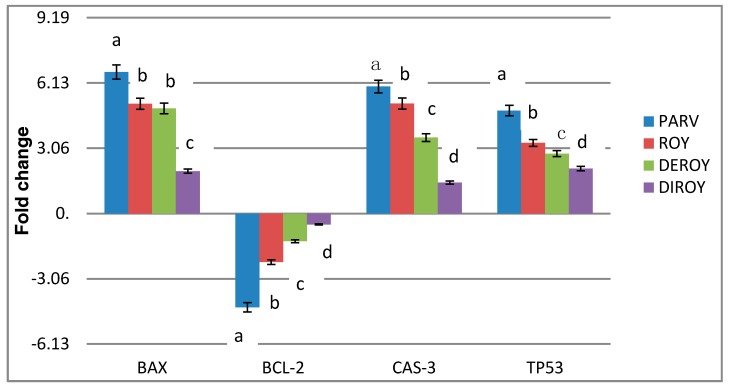
RT-PCR analysis of the mRNA expression of *TP53, Cas-3*, *Bax,* and *Bcl-2* in H7AX glioma cells following treatment with Deroy, Diroy, Roy, and Parv D for 24 h. The transcript level of each gene was normalized to the expression of a reference gene (*18S rRNA*). Data is presented as fold change in cells treated with tested compounds versus untreated, control cells, in which the expression level of the genes was set as 1. The mean values ± SD were calculated from triplicate. The same letter at the same genes are not significantly different at the level of *P* > 0.05.

The effect of Deroy, Diroy, Roy, and Parv D treatment at IC_50_ on gene expression was also analyzed. Following 24-h treatment, the mRNA expression of *TP53, Bax,* and *Cas-3* was found to be significantly increased in the cells treated with all tested compounds, while that of *Bcl-2* was decreased (Figure 5).

### 3.5. Effect of Deroy, Diroy, Roy, and Parv D on Mitochondrial Membrane Potential Loss (ΛΨm) in Primary H7PX Glioma Cells

**Figure 6:**
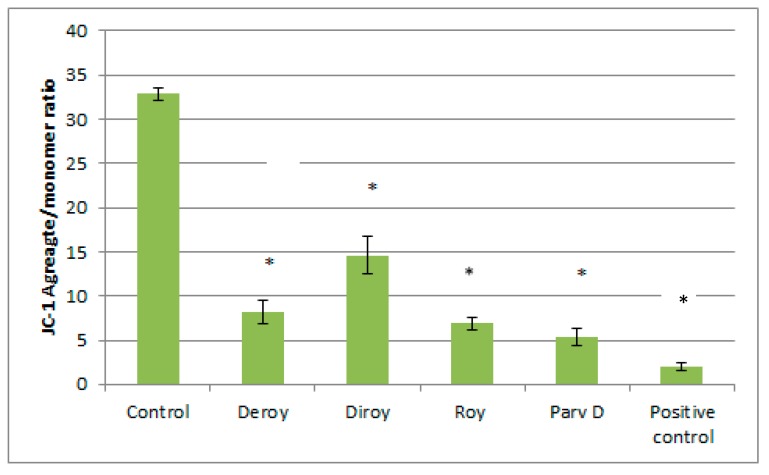
The effect of Deroy, Diroy, Roy, and Parv D on mitochondrial membrane potential in primary H7PX glioma cells. MMP is expressed as a ratio of 530 nm/590 nm to 485 nm/538 nm (aggregates to monomer) fluorescence, as quantified with a fluorescent plate reader after JC-1 staining. The data represent means ± SD. * *P* < 0.05 Control vs. Deroy, Diroy, Roy, and Parv D. Temozolomide was used as a positive control.

The mitochondrial membrane potential was measured by staining with JC-1 after treatment with IC_50_ for each of the pure compounds (Deroy, Diroy, Roy, and Parv D), to evaluate the functional status of mitochondria. After 24 h exposure, mitochondrial membrane potential was significantly decreased in primary H7AX glioma cells after treatment with all compounds compared to the control (Figure 6). Our data suggest that mitochondrial dependent mechanism contributed to abietane diterpenes (Deroy, Diroy, Roy, and Parv D) mediated apoptosis in primary H7AX glioma cells by mitochondrial membrane depolarization.

## 4. Discussion

Although the plant kingdom constitutes an extraordinary reservoir of novel bioactive organic molecules, this resource was not fully appreciated until the recent worldwide surge of interest in herbal medicine [24]. Medicinal plants can biosynthesize chemical compounds with different biological properties [25], and these have long been used for treating various civilization diseases including cancer [26,27,28,29]. Cancer is an established global health problem known to account for nine million deaths globally, and is expected to increase to about 15 million by 2030 [27]. Therefore, there is a pressing need to identify new natural compounds with potential anti-cancer activity. The biological studies revealed that abietanes are active and Parv D is the most active compound. Parv D has a very apolar feature on the positions C-6 and C-7, like Deroy, which also has an apolar part on the same C-6 and C-7 positions. Roy follows this tendency in the C-6 and C-7 polarity and bioactivity. The compound Diroy is the less bioactive abietane and this is in agreement with the more polar feature on the C-6 and C-7 positions. This polar structure-activity relationship could be explained due to a better membrane interaction [11,12,13,14,15,16,17].

Many studies have shown that plant extracts or pure compounds isolated from plants are able to induce apoptosis in various types of cancer [22,30,31,32]. Although revolutions in the modern drug industry have given rise to a trend of replacing herbal remedies with modern drugs, studies of natural compounds from herbal plants and their activities are still of interest for drug discovery; they are not only characterized by better absorption but also by lower toxicity than chemical drugs. Around 60% of drugs in current use, including anti-cancer drugs, are of plant origin. Of these, the abietane diterpenes have demonstrated strong biological activity, particularly with regard to their cytotoxic, anti-proliferative and antimicrobial potential [17,32,33]. Therefore, in recent years, many molecular studies have examined their influence on various cancer cells [15,19].

The present study describes the first evaluation of the potential of pure 6,7-dehydroroyleanone (Deroy), 7β,6β-dihydroxyroyleanone (Diroy), 7α-acetoxy-6β-hydroxyroyleanone (Roy), and parvifloron D (Parv D) isolated from *P. madagascariensis* and *P. ecklonii* to induce apoptosis in a primary glioma cell line. These members of the Lamiaceae family are particularly interesting due to their high content of secondary metabolites, including abietane diterpenes [6].

Apoptosis is an important process because it involves several biochemical and morphological changes that eventually lead to cell death [34]. The most frequently-mutated genes, independent of those giving rise to the tumor, are the *TP53* DNA damage checkpoint tumor suppressor genes [35]. The initiation of apoptosis itself is also dependent on the balance of two apoptosis regulators, *Bcl-2* and *Bax*: A balance needs to be maintained between *Bcl-2* and *Bax* expression to ensure cell survival [36], with higher *Bcl-2* expression resulting in the inhibition of apoptosis [37,38]. Current anticancer therapies, such as chemotherapeutic agents and radiotherapy, act by inducing apoptosis in various cancer cells [39]. Literature reports suggest that the common initial event in the majority of apoptotic processes involves DNA damage or damage to various other critical molecules [40,41].

The four compounds tested in the present study were found to induce apoptosis and G2/M cell cycle arrest, and increase phosphorylated H2A.X levels, by influencing the expression of pro- and anti-apoptotic genes in the H7PX glioma cell line. Of these four compounds, Deroy, Roy, and Parv D displayed stronger anticancer activity than Diroy. These findings represent the first example of apoptosis induction in a H7PX glioma cell line.

Other preliminary studies demonstrate that the same tested compounds (6,7-dehydroroyleanone, 7α-acetoxy-6β-hydroxyroyleanone and parvifloron D) isolated from *P. madagascariensis* and *P. ecklionii* induced apoptosis in CCRF-CEM and A549 cell lines by damaging DNA, increasing ROS levels and mitochondrial copy number, decreasing mitochondrial membrane potential and by changing the level of expression of pro and anti-apoptotic genes (manuscript in draft).

Not only did Parv D demonstrate the greatest cytotoxic effect against the tested H7PX line in the present study, it also displayed the strongest cytotoxic activity against CCRF-CEM and A549 cell lines in other experiments (manuscript in draft). Similarly, Burmistrova et al. report that Parv D displays cytotoxic activity against various leukemia cell lines, including HL-60, U-937, MOLT-3, and K-562 [6]. They propose that the mechanism of action is associated with a decrease in mitochondrial membrane potential and the release of cytochrome c, and that it may be amplified by inhibition of extracellular signal-regulated kinases (ERKs) 1/2 signaling; they also suggest it may be caused by a mechanism dependent on intracellular ROS generation in leukemia cells [19]. Santos-Rebelo et al. also report that Parv D obtained from *P. ecklonii* displayed a cytotoxic effect on pancreatic cell lines [42]. In the present study, it was found that of the four tested compounds, Parv D treatment induced the highest level of phosphorylated histone. It has been noted that increased levels of phosphorylated H2A.X are indicative of the presence of DSBs, which have been proposed as a cause of apoptosis induction in cancer cells [43]. In a previous study, Garcia et al. also reported that Parv D increased the level of mtDNA damage in a CCRF-CEM cell line [15].

While the H7PX glioma cells tested in the present study were found to be resistant to Diroy treatment across the tested range of concentrations, with cell viability scores of 80–100% being observed, the CCRF-CEM and A549 lines tested elsewhere were far more sensitive (manuscript in draft). We suspect that the difference observed in the potential of the four tested compounds may be attributed to a combination of differences in their chemical structures (functional groups) and the sensitivities of the cancer cells. Garcia et al. report that Deroy obtained from *P. madagascariensis* is able to induce apoptosis by triggering the intrinsic cell death pathway in cancer cells through the activation of caspases-3 and caspases-9 [15]. From these promising results, three of the tested compounds demonstrate strong cytotoxic effects on the H7PX glioma human cell line; however, more research based on molecular chemistry, molecular docking analysis and ideally, in vivo studies, is needed to clarify their interactions with cellular components.

## 5. Conclusions

Pure parvifloron D, 7α-acetoxy-6β-hydroxyroyleanone and 6,7-dehydroroyleanone isolated from *P. madagascariensis* and *P. ecklonii* may exhibit anticancer effects in H7PX glioma cell line by inducing apoptosis, G2/M cell cycle arrest and DSBs (double strand breaks), indicated by elevated phosphorylated H2AX, decreasing mitochondrial membrane potential, and by changing the level of expression of pro and anti-apoptotic genes. These natural compounds may hence prove beneficial in the treatment of different cancer types. We hope that our discoveries may contribute to the development of new anticancer treatments, but further in vivo evaluation is urgently required.
